# Supplementary material for: Vitamin D Modified DSS-Induced Colitis in Mice via STING Signaling Pathway
Source: Biology (Basel). 2025 Jun 18;14(6):715. doi: 10.3390/biology14060715 (PMC12190092; doi:10.3390/biology14060715)
Supplement: Supplementary file 1 [file biology-14-00715-s001.zip › Supplementary Materials S1: 16s-report of gut microbiota/Groups/heatmap/nogroup/cluster_norm_100log10.pdf]

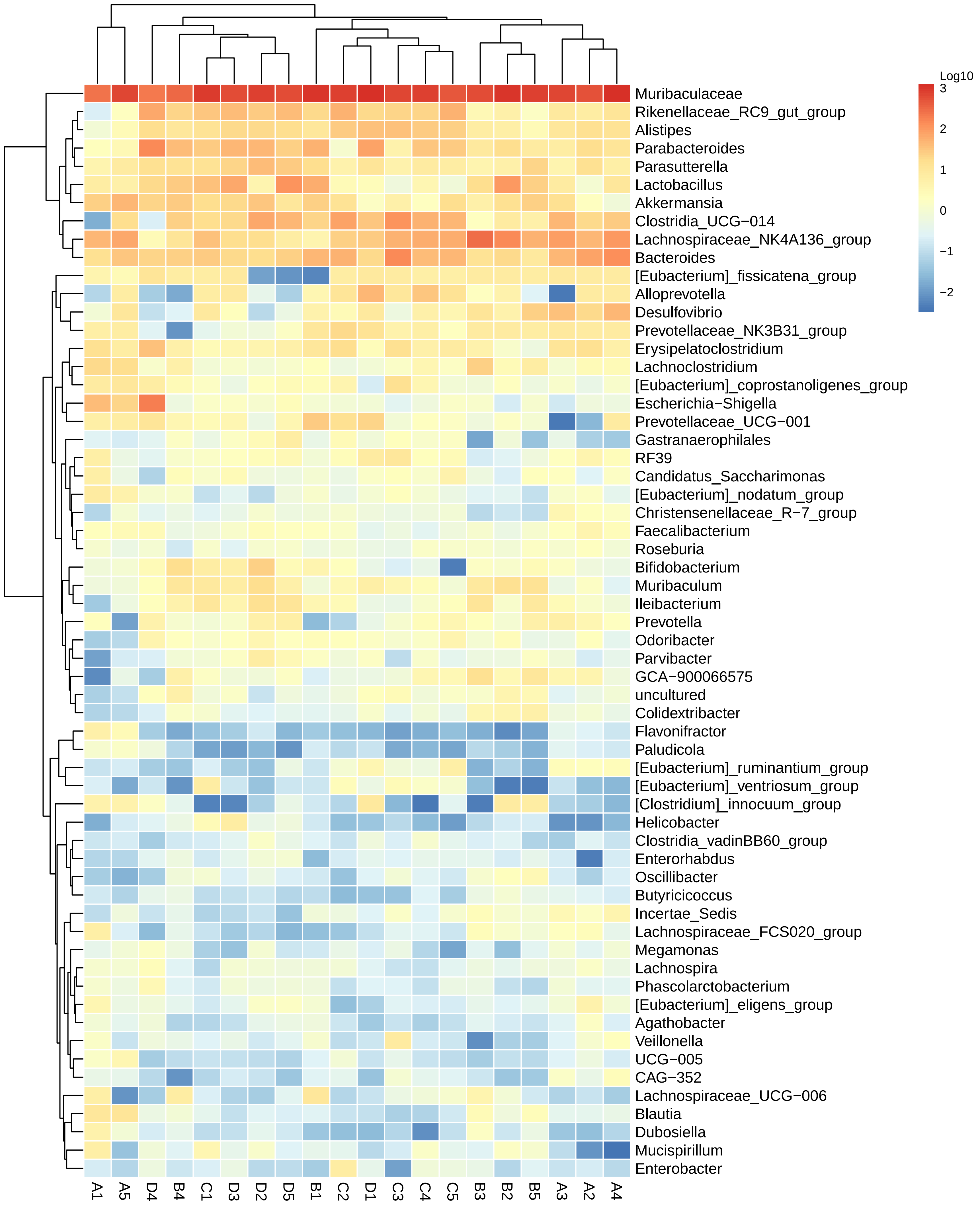
Log10
3
2
1
0
−1
−2
Muribaculaceae
Rikenellaceae_RC9_gut_group
Alistipes
Parabacteroides
Parasutterella
Lactobacillus
Akkermansia
Clostridia_UCG−014
Lachnospiraceae_NK4A136_group
Bacteroides
[Eubacterium]_fissicatena_group
Alloprevotella
Desulfovibrio
Prevotellaceae_NK3B31_group
Erysipelatoclostridium
Lachnoclostridium
[Eubacterium]_coprostanoligenes_group
Escherichia−Shigella
Prevotellaceae_UCG−001
Gastranaerophilales
RF39
Candidatus_Saccharimonas
[Eubacterium]_nodatum_group
Christensenellaceae_R−7_group
Faecalibacterium
Roseburia
Bifidobacterium
Muribaculum
Ileibacterium
Prevotella
Odoribacter
Parvibacter
GCA−900066575
uncultured
Colidextribacter
Flavonifractor
Paludicola
[Eubacterium]_ruminantium_group
[Eubacterium]_ventriosum_group
[Clostridium]_innocuum_group
Helicobacter
Clostridia_vadinBB60_group
Enterorhabdus
Oscillibacter
Butyricicoccus
Incertae_Sedis
Lachnospiraceae_FCS020_group
Megamonas
Lachnospira
Phascolarctobacterium
[Eubacterium]_eligens_group
Agathobacter
Veillonella
UCG−005
CAG−352
Lachnospiraceae_UCG−006
Blautia
Dubosiella
Mucispirillum
Enterobacter
A1
A5
D4
B4
C1
D3
D2
D5
B1
C2
D1
C3
C4
C5
B3
B2
B5
A3
A2
A4
